# Factors influencing injury severity score regarding Thai military personnel injured in mass casualty incident April 10, 2010: lessons learned from armed conflict casualties: a retrospective study

**DOI:** 10.1186/1471-227X-12-1

**Published:** 2012-01-03

**Authors:** Nuttapong Boonthep, Suthee Intharachat, Tassanee Iemsomboon

**Affiliations:** 1 Department of Trauma and Emergency Medicine, Phramongkutklao Hospital, Bangkok, Thailand

## Abstract

**Background:**

Political conflicts in Bangkok, Thailand have caused mass casualties, especially the latest event April 10, 2010, in which many military personnel were injured. Most of them were transferred to Phramongkutklao Hospital, the largest military hospital in Thailand. The current study aimed to assess factors influencing Injury Severity Score (ISS) regarding Thai military personnel injured in the mass casualty incident (MCI) April 10, 2010.

**Methods:**

A total of 728 injured soldiers transferred to Phramongkutklao Hospital were reviewed. Descriptive statistics was used to display characteristics of the injuries, relationship between mechanism of injury and injured body regions. Multiple logistic regressions were used to calculate the adjusted odds ratio (adjusted OR) of ISS comparing injured body region categories.

**Results:**

In all, 153 subjects defined as major data category were enrolled in this study. Blast injury was the most common mechanism of injury (90.2%). These victims displayed 276 injured body regions. The most common injured body region was the extremities (48.5%). A total of 18 patients (11.7%) had an ISS revealing more than 16 points. Three victims who died were expected to die due to high Trauma and Injury Severity Score (TRISS). However, one with high TRISS survived. Factors influencing ISS were age (p = 0.04), abdomen injury (adjusted OR = 29.9; 95% CI, 5.8-153.5; *P *< 0.01), head & neck injury (adjusted OR = 13.8; 95% CI, 2.4-80.4; *P *< 0.01) and chest injury (adjusted OR = 9.9; 95% CI, 2.1-47.3; *P *< 0.01).

**Conclusions:**

Blast injury was the most common mechanism of injury among Thai military personnel injured in the MCI April 10, 2010. Age and injured body region such as head & neck, chest and abdomen significantly influenced ISS. These factors should be investigated for effective medical treatment and preparing protective equipment to prevent such injuries in the future.

## Background

In the past two years, frequent mass casualty incidents (MCIs) stemming from political conflicts have occurred in Bangkok, Thailand. The first occurred October 7, 2008 and the second April, 2009 when Phramongkutklao's emergency rescue teams were activated in a local emergency response system. However, no published study has reported these MCIs. This study investigated the MCI stemming from political conflict April 10, 2010. This political conflict deviated from peaceful protest to metropolitan riotousness and had different characteristics from the past such as weapons of mass destruction were used by unknown forces leading to military MCI. Fortunately, in this event, integration of each army medical support unit merging with civilian medical services ensured provision of comprehensive care for all casualties. Prehospital treatment received cooperation from many government sectors including the Ministry of Defence that prepared field-operation military medical teams to transport injured soldiers to Phramongkutklao (PMK) Hospital, the main military level 1 trauma center in the Bangkok metropolis.

MCI, according to The Joint Commission on Accreditation of Healthcare Organization (JCAHO), describes the event when healthcare needs exceed resources that requiring extraordinary resources from every departments in hospital or requiring referral to other hospitals. Emergency medicine physicians, who specialize in disaster medicine, serve important roles as chiefs of rescue teams in response to MCI according to the hospital response plan as follows: START triage which involves screening patients related to their severity, perform initial treatment, cooperate with other specialists and distribute patients to a specific department in order to receive further definitive care [1-5].

A previous study of MCI regarding Thai military units in the southern trauma registry reported mechanism of injury; 71% by blast and 29% by firearm or gunshot wound (GSW) [6]. Explosions and firearms differ in the injured body region, distribution of severity, length of stay and inpatient death. Knowledge of mechanism and distribution of injuries are important keys for proper medical treatment and preventive measures [6-9].

Injury Severity Score (ISS) is an anatomical scoring system for patients with multiple injuries. This score has served as the standard summary measure of anatomical injury for more than 20 years. The cut-off value of ISS more than 16; shows 98.5% for sensitivity and 99.9% for negative predictive value. ISS not only is simple to use, but also has a high specificity of about 99.8% in prediction of mortality [10-16].

The main purpose of this study was to reveal the factors influencing ISS in Thai military personnel and report mechanism of injury as well as distribution of injured body regions for effective medical treatment and preparing military protection gear in the future.

## Methods

### Study design

In this retrospective study, the medical records of all injured Thai military personnel in MCI April 10, 2010, treated in PMK Hospital, were reviewed. Demographic data of patients and the nature of injuries were obtained from the medical records and PMK trauma registry major data collection form as shown in additional file 1.

ISS was classified according to the Abbreviated Injury Scale 2005 (AIS 2005) following the guidelines of the Association for the Advancement of Automotive Medicine (AAAM); an international multidisciplinary organization for crash injury control [17]. Injured body regions were classified in six regions as follows: head & neck, face, chest, abdomen, extremities and external body region. Numbers of injuries were recorded according to body regions with the agreement that multiple wounds in one region were counted as one injury, with a described definition in detail as shown in additional file 2.

### Study patients

Assessment factors, correlated with the ISS in Thai military personnel injured in MCI, require identification of the total number of traumatized population. Employing a sampling group is likely to reduce significant bias.

The traumatized population was categorized by severity of injury into PMK’s major and minor data categories as described below; then included only major data category for analysis because this group represented a high severity of injuries and used proper category to reveal which factors influenced ISS. Finally, major data category for the group, comprising a total of 153 subjects, was sent for analysis.

Inclusion criteria included;

1. Injury in MCI April 10, 2010

2. Criteria diagnosis of PMK Hospital’s major data category included at least one parameter below:

2.1 Injury to more than one body region

2.2 Any skeleton or internal organ injury of the head, neck, chest, abdomen or extremities (including fractured ribs)

2.3 Any loss of consciousness

2.4 ISS ≥ 16

2.5 Death following injury

2.6 Burns (> 20% body surface area or airway burns)

2.7 Undergoing trauma laparoscopy, laparotomy or diagnostic peritoneal lavage

2.8 Being intubated prehospital or in emergency department

2.9 Admission to intensive care unit

2.10 Fracture tibia/fibula above ankle level

Exclusion criteria

1. Criteria diagnosis of PMK Hospital’s minor data category, isolated injury to one body region, specified below:

1.1 Upper limb closed fracture/dislocation at or below level of neck of humerus

1.2 Lower limb closed fracture/dislocation at or below level of the ankle

1.3 Isolated closed fracture of fibula or patella

1.4 Soft tissue injury include partial or complete amputation of a digit

1.5 Isolated tendon injury

1.6 Minor burns (< 20% body surface area)

1.7 Isolated mandibular fracture

1.8 Minor scalp contusion or laceration with no neurological signs

### Outcomes

The objective of this study was divided into primary and secondary outcomes. The primary outcome was used to identify factors influencing the ISS regarding Thai military personnel injured in MCI April 10, 2010. Secondary outcome was used to describe the mechanism of injury and distribution of injured body regions.

### Ethical statement

The Ethics Research Committee of the Royal Thai Army Medical Department approved the study (R089h/53). STROBE guidelines, for reporting observational study, were utilized in the drafting of this report [18].

### Statistical analysis

Descriptive statistics was used to display characteristics of the injuries, relationship between mechanism of injury and injured body regions. Chi-square test was used to assess significance of coefficient. Multiple logistic regressions were used to calculate the adjusted odds ratio (adjusted OR) of ISS comparing injured body region categories.

## Results

The MCI occurred April 10, 2010 in Bangkok where crowds, controlled by law enforcement officers, created political conflict. Security forces' attempts to disperse these red-shirt protesters resulted in confrontations and clashes in several spots earlier in the afternoon. The first clash took place at about 2 pm when hundreds of protesters from Phan Fa Bridge went to the First Army Area of Royal Thai Army but this event could still be controlled.

**Figure 1:**
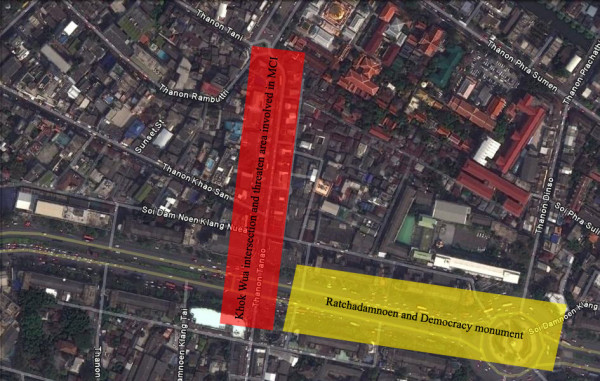
**Map demonstrating MCI April 10, 2010**. Red bar demonstrates most critical area in this MCI, while yellow bar shows second most common area occurred in MCI.

The second clash took place at about 7 pm when red-shirt protesters stepped up their struggle in the night, firing grenades and bullets into security forces, drawing a response with live rounds at the Khok Wua Intersection near the Democracy Monument as shown in Figure 1.

Finally, 20 people died from armed conflict casualty in Saturday's violence, including four soldiers, fifteen red shirts and one photojournalist. The injured Thai military personnel from armed conflict were transferred to PMK Hospital located about 5.2 kilometres northeast of the scene.

A total of 728 injured soldiers were transferred to PMK Hospital and the author enrolled 153 subjects grouped in major data category in the study, involving 276 injured body regions. The author also emphasized that one person may have had more than one injured body region.

All of the victims were male and their ages ranged from 19 to 55 years old; mean age was 27.4 ± 9 years old. Blast injury was the most common mechanism of injury affecting 90.2% in this MCI. The second most common mechanism of injury was firearm injury accounting for 6.5% and personal assault accounting for 3.3% of the MCI. Most MCI occurred at Khok Wua Intersection (75.2%) as shown in Figure 1. About 29% of the Thai military personnel were transported to PMK Hospital within the first hour after injury as shown in Table 1.

**Table 1:** Characteristics data of Thai military personnel injured in MCI

Parameter	ISS category		Overall (N = 153)	*P *value^§^
			
	ISS < 16 (n = 135)	ISS ≥ 16 (n = 18)		
**Age group (years)**				0.04
Below 25	88	8	96	
25-34	21	6	27	
35-44	7	3	10	
45 and older	19	1	20	

**Mechanism of injury**				0.69
Personal assault	5	0	5	
Gunshot	9	1	10	
Blast	121	17	138	

Prehospital time (minutes)*				0.29
≤ 30	19	1	20	
31-60	19	5	24	
61-720	59	6	65	
> 720	25	5	30	

* Missed prehospital time of 14 from 153 cases due to unreliable accuracy of time record, the remaining accurate prehospital time was 139 cases

^§ ^Considered to be statistically significant when *P *value < 0.05

**Figure 2:**
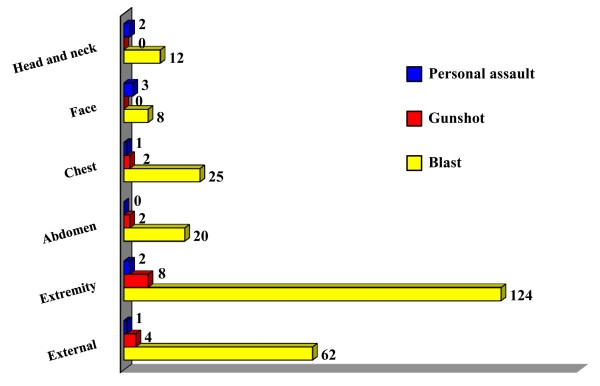
**Mechanism of injury and injured body regions among Thai military personnel in MCI**. X-axis represents the number of injured body regions, Y-axis represents each body region. *For example the extremity region had a total 134 injured body regions composed of 124 injured body regions from blast injury, 8 injured body regions from gunshot, and 2 injured body regions from personal assault.

The most common injured body region was the extremities, 48.5% (from blast injury, 90.2%, firearm, 6.5% and personal assault, 3.3%). Reports showed a high percentage of injuries on extremities and external region especially from blast injury. On the other hand, a low percentage in all mechanism of injuries affected the head & neck, chest, face and abdomen regions as shown in Figure 2.

Those with high ISS, more than 16 points, totalled 18 of 153 victims (11.8%). Three subjects who died were expected to die due to high TRISS. However, one with high TRISS survived.

The factors influencing ISS at a statistically significant difference at the 0.05 level were age (p = 0.04), abdomen injury (adjusted OR = 29.9; 95% CI, 5.8-153.5; *P *< 0.01), head & neck injury (adjusted OR = 13.8; 95% CI, 2.4-80.4; *P *< 0.01) and chest injury (adjusted OR = 9.9; 95% CI, 2.1-47.3; *P *< 0.01) as shown in Tables 1 &2.

**Table 2:** The multiple logistic regressions model for ISS comparing each injured body region categories

Parameter	Adjusted OR	95% CI	*P *value^§^
**Head and neck injury**	13.8	(2.4-80.4)	< 0.01
**Face injury**	5.1	(0.6-40.4)	0.12
**Chest injury**	9.9	(2.1-47.3)	< 0.01
**Abdomen injury**	29.9	(5.8-153.5)	< 0.01
**Extremity injury**	0.9	(0.2-4.4)	0.88
**External injury**	1.8	(0.5-6.9)	0.37

^§ ^Considered to be statistically significant when *P *value < 0.05

## Discussion

Over the past ten years, the military MCI has been confined in specific areas, particularly the three southern border provinces where terrorism produces continuing violence with a variety of incidents primarily shootings, and secondly, bombings both in rural and downtown areas. A previous study of military MCI regarding Thai military units in the southern trauma registry reported that mechanism of injury about 71%, blast injury and 29%, firearm or gunshot wound [6]. This present study showed a higher ratio of blast injury (90.2%) while the second most common injury was gunshot wound (6.5%) implying that weapons of mass destruction (WMDs) will be one of the major concerns in our armed conflict casualties in the future even though the incident was in the capital city.

The previous study of southern conflict in Thailand demonstrated the anatomic distribution of injured body regions indicating head & neck was 21.8%, the torso (chest, abdomen, trunk and pelvis) was 24.5% and the most common injured body region was the extremities 51.6% [6]. Compared with a previous study, this represented a lower distribution, i.e., head & neck (5.1%), abdomen (7.9%) and chest (10.1%). Perhaps this is due to effective protective body armour vests and helmets. However, the injury to the extremities still exhibits a high percentage, 48.5% (134 of 276 injured body regions) implying that protection in this areas is not effective enough.

In this study, prehospital treatment received cooperation from many government sectors and the Ministry of Defence to prepare field-operation military medical teams to transport injured soldiers to PMK Hospital where prehospital time was recorded by military health care officers. Although this MCI occurred April 10, 2010, many injured soldiers had to be transported at the same time, leading to unreliable accuracy of time recordings. Unreliable prehospital time data was found in 14 of 153 cases, so the prehospital time records of the remaining 139 cases were analyzed for accuracy. About 29% of injured soldiers presented to the hospital within the first hour of trauma care that may be inappropriate in prehospital transportation during this MCI because health care providers could not suddenly evacuate casualties during continuous firing and bombing in those dangerous areas and transportation was blocked by crowds.

The analysis finally showed that the factors influencing ISS with a statistically significant difference at the 0.05 level were age (p = 0.04), abdomen injury (adjusted OR = 29.9; 95% CI, 5.8-153.5; *P *< 0.01), head & neck injury (adjusted OR = 13.8; 95% CI, 2.4-80.4; *P *< 0.01) and chest injury (adjusted OR = 9.9; 95% CI, 2.1-47.3; *P *< 0.01).

This study emphasized report only MCI April 10, 2010. Soldiers with high ISS, more than 16 points, totalled 18 of 153 victims (11.8%). This low percentage of severe injury is the characteristics of this MCI; the protective equipments, that lower ISS, may be effectively used.

These data including mechanism of injury & distribution of injured body regions and factors influencing ISS were important keys in the implications for hospital organizations to manage limited health care resources. As a result, management in MCI could be handled within the resources of the emergency team based on emergency physicians cooperating with other specialists, as well as nursing staffs. Finally, it was found that three victims were predicted to die due to high TRISS but one victim unexpectedly survived despite having a high TRISS due to effective resuscitation and good cooperation from multidisciplinary health care services.

### Limitation

By nature, research on disaster medicine is largely descriptive as MCI is virtually impossible to study via prospective randomized controlled trials and the study could not be double blinded or concealed.

Regarding hospital preparedness in specific circumstances as military MCI, health care providers cannot normally access in the operation zone where WMDs were used and could not normally evacuate or transport casualties because of entrapment by the crowds resulting in delayed prehospital time from minutes to hours or even days.

### Implementation and suggestion

Knowledge in mechanism of injury, distribution of injured body regions together with the proven factors influencing ISS used to predict mortality, are all important keys for proper medical management and preventive measures.

Implications concerning hospital organizational aspects include improving management with limited health care resources and enhancing hospital surge capacity for MCI. Implications concerning the Ministry of Defence aspects include improving effectiveness of protective equipment in future military MCI. Implications concerning the national aspects include establishing harmonized military-civilian collaboration in MCI response network.

This study is based on cases in a military hospital, and recommendations may require non- military studies in public health hospitals to compare results. MCIs are heterogeneous by nature and their unexpectedness favors an "all-hazard" approach including radiation & nuclear wastes, bioterrorism, chemical weapons or explosion. MCI preparedness must be prompt every time.

## Conclusions

Blast injury was the most common mechanism of injury among Thai military personnel injured in the MCI April 10, 2010. Age and injured body regions such as head & neck, chest and abdomen were proven factors influencing ISS. These factors should be considered for effective medical treatment and preparing protective equipment to prevent such injuries in the future.

## Competing interests

The authors have no relevant financial interests, financial relationships, or competing interests (financial or nonfinancial) to report.

## Authors' contributions

NB reviewed literature, conceived & designed the study, requested & revised proposal submission throughout ethical process, managed the collected data, performed the data analysis & result interpretation, drafted & revised the manuscript. SI conceived the study, participated to the study design. TI supervised the data collection, carried out the chart review, valuable help in data coding, managed the collected data. All authors have read and approved the final manuscript.

## Pre-publication history

The pre-publication history for this paper can be accessed here:

http://www.biomedcentral.com/1471-227X/12/1/prepub

## Supplementary Material

Additional file 1 **Data Collection Form in this Research**. Click here for file

Additional file 2 **Definition in this study**. Click here for file
