# Supplementary material for: Factors influencing injury severity score regarding Thai military personnel injured in mass casualty incident April 10, 2010: lessons learned from armed conflict casualties: a retrospective study
Source: BMC Emerg Med. 2012 Jan 3;12:1. doi: 10.1186/1471-227X-12-1 (PMC3276435; doi:10.1186/1471-227X-12-1)
Supplement: Additional file 1 — Data Collection Form in this Research. [file 1471-227X-12-1-S1.PDF]

Additional file 1

Data Collection Form in this Research

PMK Trauma Registry Major Data Collection Form

General data (Personal data use running number)

Injury data

Date and time …………………………………………..

Mechanism ☐Blunt ☐Penetrating

Pre-hospital

At scene………………...Departing scene………………At hospital……………………….

Time assessment……….Pulse rate…….. Respiratory rate……… BP………

E.…V.…M….

Cardiopulmonary Resuscitation……….. Fluid type……… Medication………………

Referring hospital

Arrival date……………..Arrival time……………………...

Time assessment……….Pulse rate…….. Respiratory rate……… BP………

E.…V.…M….

Cardiopulmonary Resuscitation……….. Fluid type……… Medication………………

Emergency department admission

Arrival date……………..Arrival time……………………...

Time assessment……….Pulse rate…….. Respiratory rate……… BP………

E.…V.…M….

Cardiopulmonary Resuscitation……….. Fluid type……… Medication………………

Investigation ☐ CT (Computed Tomography) Time in……… Time out ……….........

☐ DPL (Diagnostic Peritoneal Lavage)

☐ FAST (Focused Assessment with Sonography for Trauma)

☐ Other

Emergency department disposition…………….. To department ……………………………

Name of hospital (if transferred)…………………

Operation

1. ……………………………… date …………. time ………..

description ………………

2. ……………………………… date …………. time ………..

description ………………

3. ……………………………… date …………. time ………..

description ………………

4. ……………………………… date …………. time ………..

description ………………

Diagnosis

1. ……………………………………………..
2. ……………………………………………..
3. ……………………………………………..
4. ……………………………………………..

Score: ISS ………….. RTS ………………TRISS ………………

<u>Performance indicators</u>

Pre-hospital

☐ Endotracheal tube intubation if GCS $\leq$ 8 (GCS=Glasgow Coma Score)

☐ Scene time < 20 minutes

☐ IV cannula if BP < 90/60

Resuscitation

☐ Endotracheal tube intubation within <10 minutes if GCS ≤ 8

☐ Exploration of penetrating trauma <1 hour of arrival if have indication

☐ <3 hour admit

☐ Performing head CT if GCS <13

☐ Patient goes to CT <1 hour if indication to perform CT

☐ Performing CXR if presenting multiple injuries

☐ Performing blood transfusion if blood loss > 2000 ml.

Definitive care

☐ Performing dislocation joint reduction <1 hour of arrival

☐ Missed fracture < 24 hours

☐ Thromboembolic prophylaxis < 24 hours

☐ Fracture fixation < 24 hours of arrival

☐ Time from injury to craniotomy

Review

☐ Ischemia limb < 4 hours of injury

☐ Unplanned operation

☐ Unplanned transfer to ICU

☐ All injuries diagnosed

Time from injury to laparotomy ………………

Injury outcome

Day in ICU …………………… Day in ward ………………………

Discharge data ……………………………………………………

Discharge status ☐ survived ☐ died

Transfer unit

Time …………………………. Hospital name (if applicable)

Death details

Place of death ……………….. Time of death ……………………………………..

Additional data items / Complications

....
