# Supplementary material for: Factors influencing injury severity score regarding Thai military personnel injured in mass casualty incident April 10, 2010: lessons learned from armed conflict casualties: a retrospective study
Source: BMC Emerg Med. 2012 Jan 3;12:1. doi: 10.1186/1471-227X-12-1 (PMC3276435; doi:10.1186/1471-227X-12-1)
Supplement: Additional file 2 — Definition in this study. [file 1471-227X-12-1-S2.PDF]

Additional file 2

Definition in this study

1) **Mass casualty incident (MCI)**

Mass casualty incident, according to The Joint Commission on Accreditation of Healthcare Organization (JCAHO), describes the event when healthcare needs exceed healthcare resources requiring extraordinary resources from every departments in the hospital or requiring referral to other hospitals.

2) **Body regions**

Body regions are classified in six regions as follows: head & neck, face, chest, abdomen, extremities and external body region. Numbers of injuries are recorded according to body regions with the agreement that multiple wounds in one region are counted as one injury.

3) **AIS (Abbreviated Injury Scale)**

The AIS is a way of describing the injury. On its own, AIS is not designed to provide any outcome prediction. It forms the basis for the ISS, which in turn forms the basis for other scoring systems for outcome prediction. It has undergone six revisions since its first description in 1971. AIS-71 considers only blunt injuries; AIS-85 introduced penetrating trauma. AIS-90 describes over 1300 individual injuries and fine-tunes the severity scores of many other injuries.

The AIS is a consensus-derived, anatomically-based system of grading injuries on an ordinal scale ranging from 1 (minor injury) to 6 (lethal injury). Scales for all anatomic regions and organs can be found at the American Association for the Surgery of Trauma Web site. AIS Manuals and CDs (2005) are available from the AAAM list of publications.

4) **Injury Severity Score (ISS)**

The ISS is an anatomical scoring system that provides an overall score for patients with multiple injuries. Each injury is assigned an AIS score and is allocated to one of six body regions (head & neck, face, chest, abdomen, extremities, and external structures). Only the highest AIS score in each body region is used.

The three most severely injured body regions have their score squared and added together to produce the ISS score (ISS = sum of squares of the highest AIS grade in the three most severely injured body regions). It is important to note that only one injury per body region is allowed.

The ISS ranges from 1 to 75, and an ISS of 75 is assigned to anyone with AIS of 6. Further, the definition of a patient with multiple injuries is one with an ISS greater than or equal to 16 and these require care in a designated trauma centre. An example of ISS calculation is shown in **Table 1**.

Table 1. Example calculation of Injury Severity Score

Region	Injury description	AIS*	Square top three
Head & neck	Cerebral contusion	3	**9**
Face	No injury	0	
Chest	Flailed chest	4	**16**
Abdomen	Minor contusion of liver	2	
	Complex rupture spleen	5	**25**
Extremity	Fractured femur	2	
External	No injury	0	
**Injury Severity Score**			**50**

*AIS represent Abbreviated Injury Scale

5) **Revised Trauma Score (RTS)**

The RTS, introduced in the early 1980s, is one of the most commonly used physiologic scores. It employs three specific physiological parameters; the GCS, systemic blood pressure (SBP) and the respiratory rate (RR). These parameters are coded from 0 to 4 based on the magnitude of physiological derangement, seen detail in **Table 2**.

Table 2. Coding variables for the Revised Trauma Score

GCS	SBP	RR	Coded value
13-15	> 89	>29	4
9-12	76-89	10-29	3
6-8	50-75	6-9	2
4-5	1-49	1-5	1
3	0	0	0

GCS represent Glasgow Coma Scale, SBP represent systolic blood pressure, RR represent respiratory rate

The RTS is calculated by adding together the coded values for each of these three physiological parameters. When used for field triage, the unweighted RTS determined by simply combining the coded values ranges from 0 to 12 and is calculated very easily. A score of less than 11 is considered an indication for transfer to a dedicated trauma centre. When used for quality assurance and outcome prediction, a coded form of the RTS is more often used. The coded RTS is calculated as shown next where SBPc, RRc and GCSc represent the coded (c) values of each variable: **RTSc = 0.7326*SBPc + 0.2908*RRc + 0.9368*GCSc**

The coded RTS allows for weighting of the individual components. It is important to note that the significant impact of traumatic brain injury on outcome has been well-emphasised in the coded RTS. Values for the RTS are in the range 0-7.8408; where 0

represents being dead and 7.8408 being normal. The RTS is heavily weighted towards the Glasgow Coma Scale to compensate for the importance of major head injury without multisystem injury or major physiological changes. A threshold coded RTS <4 has been proposed to identify those patients who should be treated in a trauma centre.

6) **Trauma and Injury Severity Score (TRISS)**

Champion and Boyd have demonstrated that the predictive capacity of any model is increased by the inclusion of additional relevant information in developing of the TRISS.

This method combines both anatomical and physiological measures of injury severity (ISS and RTS, respectively) and patient age in order to predict survival from trauma. TRISS determines the probability of survival (Ps) of a patient from the ISS and RTS using the following formula: $\mathbf{Ps = 1/ (1+e^{-b})}$

Where “b” is calculated from: b = b0 + b1*(RTS) + b2*(ISS) + b3*(Age Index)

The coefficients b0 - b3, seen detail in **Table 3**, are derived from multiple-regression analysis of the Major Trauma Outcome Study database. The Age Index is 0 if the patient is below 54 years of age or 1 if 55 years and over. The coefficients (b0 - b3) are different for blunt and penetrating trauma. If the patient is less than 15 years old, the blunt coefficients are used regardless of the actual mechanism of injury. TRISS quickly became the standard method for outcome assessment. It appears to be valid for both adult and child patients.

TRISS used to predict survival outcome which divides the patients into three groups as following;

1. Patients with TRISS> 0.5 are considered as patients with more than 50% survival; if the patients in this group died, it is considered “Preventable trauma death”, usually caused by ineffectiveness in medical care.

2. Patients with TRISS between 0.25 and 0.5; if the patients in this group died, it is considered “Potentially preventable trauma death”.

3. Patients with TRISS <0.25; if the patients in this group died, it is considered “Non-preventable trauma death”.

Table 3. Coefficients (b) use in determining survival probability in the Trauma and Injury Severity Score

Coefficients	Blunt	Penetrating
**b0**	-0.4499	-2.5355
**b1**	0.8085	0.9349
**b2**	-0.0835	-0.0651
**b3**	-1.7430	-1.1360
